# Combination of acamprosate and baclofen as a promising therapeutic approach for Parkinson’s disease

**DOI:** 10.1038/srep16084

**Published:** 2015-11-06

**Authors:** Rodolphe Hajj, Aude Milet, Damien Toulorge, Nathalie Cholet, Julien Laffaire, Julie Foucquier, Sandra Robelet, Richard Mitry, Mickael Guedj, Serguei Nabirotchkin, Ilya Chumakov, Daniel Cohen

**Affiliations:** 1 Pharnext, 11 rue des Peupliers, 92130 Issy-Les-Moulineaux, France; 2 Syncrosome, 163 avenue de Luminy, 13288 Marseille, France

## Abstract

Parkinson’s disease (PD) is a progressive neurodegenerative disorder characterised by the loss of dopaminergic nigrostriatal neurons but which involves the loss of additional neurotransmitter pathways. Mono- or polytherapeutic interventions in PD patients have declining efficacy long-term and no influence on disease progression. The systematic analysis of available genetic and functional data as well as the substantial overlap between Alzheimer’s disease (AD) and PD features led us to repurpose and explore the effectiveness of a combination therapy (ABC) with two drugs – acamprosate and baclofen – that was already effective in AD animal models, for the treatment of PD. We showed *in vitro* that ABC strongly and synergistically protected neuronal cells from oxidative stress in the oxygen and glucose deprivation model, as well as dopaminergic neurons from cell death in the 6-hydroxydopamine (6-OHDA) rat model. Furthermore, we showed that ABC normalised altered motor symptoms *in vivo* in 6-OHDA-treated rats, acting by protecting dopaminergic cell bodies and their striatal terminals. Interestingly, ABC also restored a normal behaviour pattern in lesioned rats suggesting a symptomatic effect, and did not negatively interact with L-dopa. Our results demonstrate the potential value of combining repurposed drugs as a promising new strategy to treat this debilitating disease.

Parkinson’s disease (PD) is the second most common neurodegenerative and progressive disorder after Alzheimer’s disease (AD), generally affecting people over 60 years old^1^. With the aging of the world population the importance of PD as an economic burden is becoming progressively more important. PD is characterised by dopaminergic deficiency due to the degeneration of nigrostriatal dopamine-producing neurons that are critically involved in the control of voluntary movements^2^, and by accumulation of α-synuclein in both the central and autonomic nervous systems^3^. Oxidative stress, excitotoxicity, synaptic alterations, apoptosis and inflammation are also prominent landmarks of the disease^4^. The degeneration of nigrostriatal dopaminergic neurons and the consequent loss of striatal dopamine underlie the development of the characteristic motor features of PD including rigidity, tremor, bradykinesia and postural instability^5^. The involvement of other neurotransmitter systems is responsible for non-motor symptoms that may precede, but invariably follow motor signs^6^.

Despite significant research efforts undertaken to address PD, available treatments are symptomatic and do not affect the progression of the disease^7^. Currently, the gold standard for treatment of PD is L-dopa in association with benserazide or carbidopa (referred to hereafter in the text as L-dopa), which is used as a replacement for the loss of dopamine in the brain of affected patients. Other therapeutic drugs such as dopamine agonists or monoamine oxidase inhibitors are also used to treat patients, but these interventions have not been proven to work better than L-dopa^8^. Dopamine replacement therapies are effective in treating particularly bradykinesia and rigidity, but in the case of L-dopa, are often associated with the development of motor complications^9^. Furthermore, L-dopa treatment was shown to induce oxidative stress^10^ in PD patients.

It is now common clinical practice to combine dopaminergic therapies to provide effective symptom relief and limit side effects associated with the higher doses of each agent^11^,^12^. However, the complex pathophysiological mechanisms involved in the etiology of PD remain poorly understood and this limits the discovery of both more effective symptomatic agents as well as treatments to modify its course. Indeed, the multifactorial and complex molecular nature of the disease^13^ may render it potentially resistant to mono-therapeutic interventions. In addition, it was postulated that several risk factors including aging^1^, genetic predisposition and environmental chemical exposure^14^ could contribute to the development of the disease. Thus, there is an urgent need for safe symptomatic treatments preferably combined with a disease-modifying activity.

The systematic analysis of available genetic and functional data in the literature has drawn our particular attention to the disturbance in the balance between glutamate and GABA systems for the development of pathological features prominent in PD^15^,^16^. Moreover, PD has a substantial comorbidity with dementias such as Alzheimer’s disease (AD). Indeed, similarly to AD, PD is characterized by progressive neurodegeneration, accumulation of reactive oxygen species, synaptic dysfunction and neuroinflammation. We previously demonstrated that multi-targeting strategy^17^ with drug combinations achieves a disease-modifying effect^18^ in various models of this dementia. We showed that a combination (ABC) of two approved drugs – acamprosate (ACP) and baclofen (BCL) – synergistically protected neurons *in vitro* and alleviated cognitive symptoms in *in vivo* models of AD^18^. Moreover, we provided evidence that the neuroprotective effect of ABC may be mediated through the simultaneous modulation of excitatory glutamate and inhibitory GABA systems. We demonstrated the positive action of ABC on several hallmarks of AD, through protection of neuronal cells from death and apoptosis, synapse preservation, normalisation of glutamate levels, decrease of oxidative stress and inflammation. Interestingly, all these features of AD are found to be shared with those of PD^4^.

In the present study, we argue that, due to the substantial overlap between AD and PD^4^,^19^, a polytherapeutic intervention could also be effective for PD. To this end, we tested the combination of ACP and BCL (ABC) for its effect in *in vitro* and *in vivo* models relevant to PD pathology.

## Results

### ABC synergistically protects against oxidative stress *in vitro*

**Figure 1:**
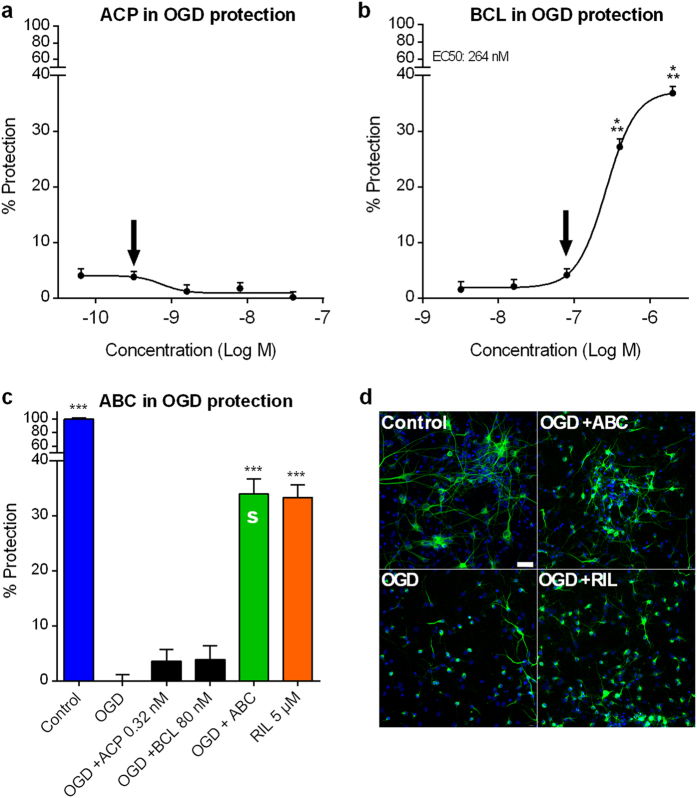
Combination of ACP and BCL acts synergistically to protect cortical neurons from oxidative stress *in vitro*. Data are derived from a combined analysis of 2 different experiments with 18 replicates per experiment. Data were normalised to the non-treated control (100%) and oxygen glucose deprived (OGD) cells (0%). Dose-response effect of ACP (**a**) or BCL (**b**) following OGD. BCL protected neuronal cells against OGD in a dose-related fashion. At inactive concentrations of individual drugs (Arrows in **(a)** and **(b)**), ABC was active with a synergistic effect (S) on cortical neurons (**c**), assessed by the Highest Single Agent approach (HSA CI = 0.12, *P*  < 2e-16), as visualised in (**d**) through immunodetection of MAP-2 (green). Experimental validation of the model was performed with Riluzole (RIL). All values are mean ± s.e.m. ***P < 0.001 versus OGD; ANOVA with Dunnett’s test. ABC: ACP 0.32 nM + BCL 80 nM. S: Synergy. Arrows (**a**,**b**) indicate the concentrations used in (**c**). Scale bar: 30 μm. Blue: Hoechst.

Oxidative stress and free radical-mediated damage, as well as modifications of energy metabolism are generally considered important components of PD pathogenesis^20^,^21^. Therefore, we tested the protective potential of ACP and BCL in the primary cortical neuronal rat model of oxidative stress in which cells were subjected to oxygen and glucose deprivation (OGD). We observed that ACP alone did not protect neurons against OGD at the tested concentrations (Fig. 1a), whereas BCL protected neuronal cells in a dose-dependent fashion (Fig. 1b). To assess the possible synergistic interaction between the two drugs, inactive doses of each compound (Fig. 1a,b arrows) were tested in combination. Riluzole, used to validate our experimental settings, reduced OGD-induced cell injury as described previously^22^. We found a significant neuronal protection from OGD with ABC in contrast to the absence of activity of individual drugs at these doses (Fig. 1c,d). This positive combination effect between ACP and BCL was confirmed by calculating the combination index (CI) using the Highest Single Agent approach^23^ (HSA CI = 0.12), providing evidence of synergism in this oxidative stress model.

### ABC synergistically protects dopaminergic neurons *in vitro* in the PD 6-OHDA-induced toxicity model

**Figure 2:**
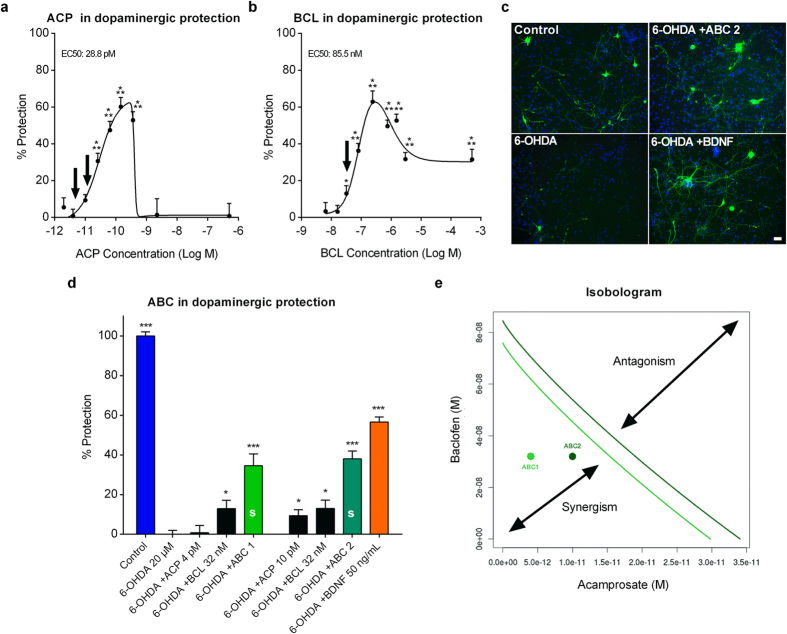
Combination of ACP and BCL acts synergistically to protect dopaminergic neurons intoxicated by 6-OHDA *in vitro*. Data are derived from a combined analysis of 5 different experiments with 18 replicates per experiment. Data were normalised to the non-treated control (100%) and 6-OHDA-intoxicated cells (0%). Dose-response of ACP (**a**) and BCL (**b**) in 6-OHDA-intoxicated mesencephalic cultures. ACP and BCL effects were dose-dependent and bell-shaped. As visualised in (**c**) through immunodetection of TH (green), ABC significantly protected dopaminergic neurons more efficiently than its individual drugs when these were used at their inactive or sub-active concentrations (Arrows in **(a)** and **(b)**), indicating their positive interaction on dopaminergic neurons (**d**). BDNF was used as a positive control. (**e**) Synergy between ACP and BCL of the combined drugs from (**a**) and (**b**), assessed by Loewe additivity model and isobologram analysis (Loewe CI = 0.60 for ABC 1 and 0.74 for ABC 2). ABC 1 (green) and ABC 2 (dark green) are each located on the left side of its respective isobole (same colour code), thus meaning that these combinations are synergistic (CI < 1). All values are mean ± s.e.m. *P < 0.05, ***P < 0.001 versus 6-OHDA; ANOVA with Dunnett’s test. ABC 1: ACP 4 pM + BCL 32 nM. ABC 2: ACP 10 pM + BCL 32 nM. S: Synergy. Arrows (**a**,**b**) indicate the concentrations used in (**c**). Scale bar: 20 μM. Blue: Hoechst.

In order to confirm the protective synergistic findings in a cellular model specific to PD, we used the *in vitro* 6-OHDA toxicity model^24^ of rat primary mesencephalic neurons. Differentiated neuronal cultures contained dopaminergic cells that were specifically identified by staining tyrosine hydroxylase (TH), the rate limiting enzyme in dopamine synthesis^25^. At low doses, individually applied ACP or BCL both protected dopaminergic neurons in a dose-dependent manner (Fig. 2a,b). Inactive or sub-active doses of ACP or BCL (arrows in Fig. 2a,b) were then combined to assess a possible synergy between drugs. We found a significantly greater protection of dopaminergic neurons in 6-OHDA-treated cultures when ABC was applied compared to the effect of each drug individually (Fig. 2c,d). This positive combination effect between ACP and BCL was confirmed by the Loewe additivity model^26^,^27^ (CI = 0.60 for ABC 1 and 0.74 for ABC 2) and isobolographic analysis (Fig. 2e). We noted that Brain-Derived Neurotrophic Factor (BDNF), used in our experiment as positive control, also reduced 6-OHDA-induced cell death as described previously^28^. These results demonstrated that the effect of the two drugs in combination was not simply additive but synergistic, which demonstrated the substantial advantage for their combination.

### ABC restores functional motor alterations *in vivo*

The synergy observed in two cellular models of PD prompted us to test ABC *in vivo* in a functional context using the 6-OHDA model^29^. The classical method of unilateral stereotaxic injection of 6-OHDA in the *substantia nigra pars compacta* (SNc) results in a massive destruction of nigrostriatal dopaminergic neurons and is largely used to model motor dysfunctions in PD^30^. When injected in the SNc, the toxic effect of 6-OHDA is well documented in terms of nigrostriatal dopaminergic cell death by oxidative stress and apoptosis^31^. Moreover, the abolition of dopamine (data not shown) and the depletion of Mazindol binding in the striatum, as well as the considerably reduced number of TH-positive neurons confirmed dopaminergic cell depletion in intoxicated rats.

**Figure 3:**
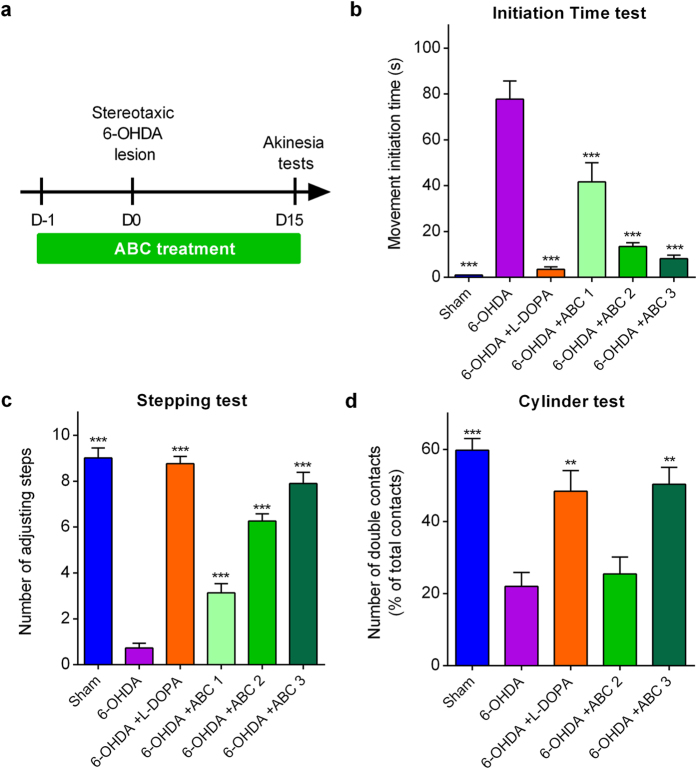
Chronic ABC treatment alleviates akinetic behaviour in 6-OHDA-lesioned rats. Stereotaxically-lesioned 6-OHDA rats compared to Sham-injected animals. Animals were chronically treated p.o. twice a day with ABC, 1 day before the lesion (D-1) until D15 where akinesia tests were done (**a**). Positive effect of ABC in the initiation time test (**b**), stepping test (**c**) and cylinder test (**d**). L-dopa was used as a reference drug. 6-OHDA or ABC did not have any effect in these behavioural tests when the control ipsilateral paw was assessed in the same rats in the initiation time and stepping tests. Values are mean ± s.e.m. Data are derived from a combined analysis performed on 2 independent experiments. For initiation time and stepping tests, *n* = 20 except for ABC 1 where *n* = 10. For the cylinder test, *n* = 10 except for 6-OHDA animals where *n* = 8 because the minimal number of contacts for 2 animals was not reached (n < 7 contacts). **P < 0.01, ***P < 0.001 versus 6-OHDA; ANOVA with Dunnett’s test. ABC 1: ACP 0.04 mg/kg + BCL 0.6 mg/kg. ABC 2: ACP 0.1 mg/kg + BCL 1.5 mg/kg. ABC 3: ACP 0.25 mg/kg + BCL 3.75 mg/kg. L-dopa: L-dopa 8 mg/kg + benserazide 12.5 mg/kg.

To assess the anti-akinetic effect of ABC, rats were chronically treated for 17 days *per os* with several doses of ABC, starting 1 day before induction of lesions by 6-OHDA (Fig. 3a). ABC dose selection in rats was based on allometric translation^32^ of effective doses observed in mouse models in our previous experiments for AD^18^. We observed that ABC significantly and dose-dependently reduced the akinetic behaviour of rats both in the initiation time and stepping tests (Fig. 3b,c). Interestingly, the highest dose (ABC 3) normalised the altered behaviour similarly to the reference treatment with L-dopa. Moreover, ABC 3 proved its significant efficacy in the test of spontaneous activity in the cylinder (Fig. 3d). Therefore, ABC treatment was able to restore a normal behaviour in 6-OHDA rats.

### ABC protects SNc dopaminergic neuronal cell bodies and striatal nerve terminals *in vivo*

**Figure 4:**
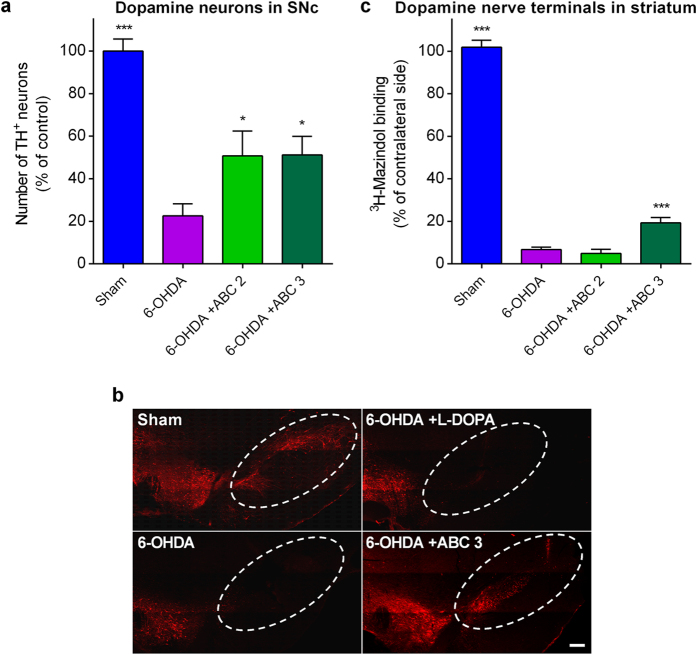
Chronic ABC treatment protects dopaminergic neuronal cell bodies and nerve terminals in 6-OHDA-lesioned rats. Stereotaxically-lesioned 6-OHDA rats compared to Sham-injected animals. Quantification of the number of dopaminergic neurons in the ipsilateral SNc (**a**) as visualised in (**b**) Through immuno-detection of TH (red). (**c**) [^3^H]-Mazindol binding in the striatum revealing dopaminergic nerve terminals. 6-OHDA or ABC did not have any effect on the number of TH^+^ neurons and on [^3^H]-Mazindol binding when the contralateral brain was assessed. Values are mean ± s.e.m. *n* = 10 per condition, except for [^3^H]-Mazindol binding where *n* = 8 for Sham animals and *n* = 9 for ABC 3. *P < 0.05, ***P < 0.001 versus 6-OHDA; ANOVA with Dunnett’s test. ABC 2: ACP 0.1 mg/kg + BCL 1.5 mg/kg. ABC 3: ACP 0.25 mg/kg + BCL 3.75 mg/kg. Scale bar: 200 μm.

We next assessed the protective potential of ABC for neuronal structures by quantifying dopamine neuronal cell bodies in the SNc and dopamine terminals in the striatum. We found that chronic ABC treatment of 6-OHDA-treated rats substantially and significantly prevented the loss of dopaminergic neurons in the SNc (Fig. 4a,b). Interestingly, we also observed that the highest dose of ABC partially but significantly protected dopaminergic nerve terminals in the striatum (Fig. 4c). Thus, anti-akinetic effects of ABC could be, at least in part, mediated by the preservation of dopaminergic neurons and their projections.

### Symptomatic treatment with ABC restores functional motor alterations in 6-OHDA pre-lesioned rats

**Figure 5:**
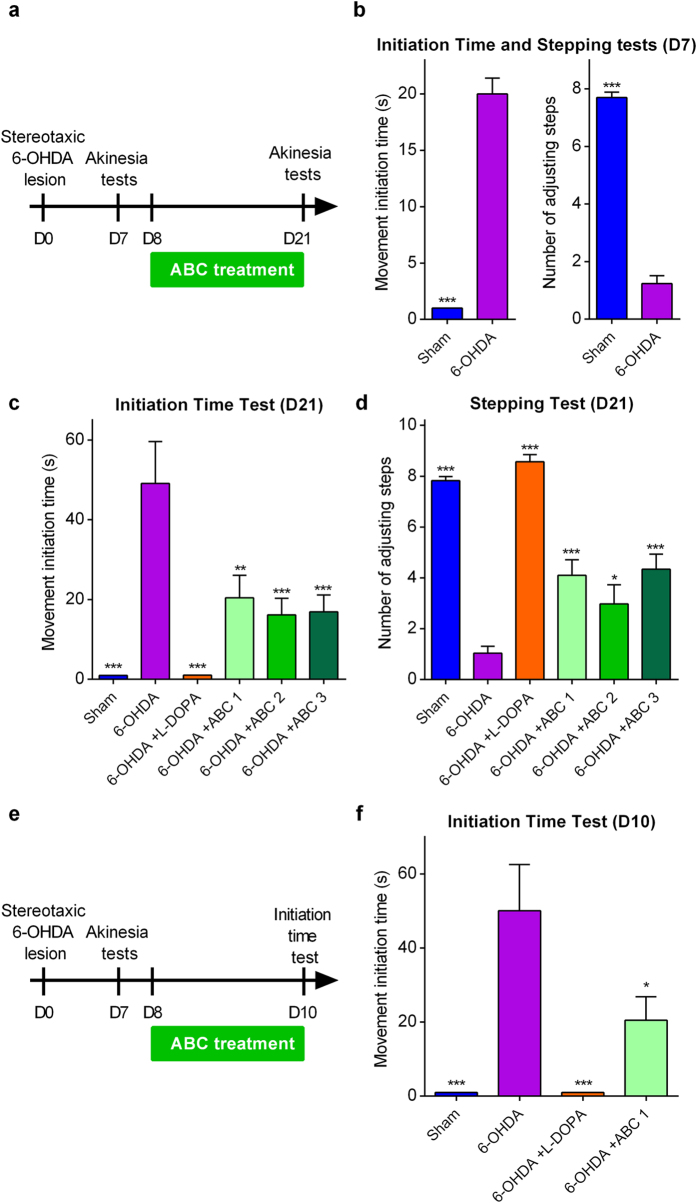
Chronic ABC treatment rapidly alleviates the symptomatic akinetic behaviour in 6-OHDA pre-lesioned rats. Stereotaxically-lesioned 6-OHDA rats compared to Sham-injected animals. Animals were chronically treated p.o. twice a day with ABC from D8 after lesion until D21 where akinesia tests were done (**a**). To check that akinetic behaviour was already manifested, initiation time and stepping tests were performed on day 7 (D7) (**b**). Positive effect of ABC at D21 in the initiation time (**c**) and stepping (**d**) tests. In a second set of experiments, animals were chronically treated p.o. twice a day with ABC from D8 after lesion until D10 (**e**) where initiation time test was done (**f**). L-dopa was used as reference drug. 6-OHDA or ABC did not have any effect in these behavioural tests when the control ipsilateral paw was assessed in the same rats in the initiation time and stepping tests. Values are mean ± s.e.m. *n* = 10 per condition. *P < 0.05, **P < 0.01, ***P < 0.001 versus 6-OHDA; ANOVA with Dunnett’s test. ABC 1: ACP 0.04 mg/kg + BCL 0.6 mg/kg. ABC 2: ACP 0.1 mg/kg + BCL 1.5 mg/kg. ABC 3: ACP 0.25 mg/kg + BCL 3.75 mg/kg. L-dopa: L-dopa 8 mg/kg + benserazide 12.5 mg/kg.

The possible anti-akinetic effect of ABC could also be due to its ability to exert a rapid symptomatic effect. To test this hypothesis, we changed the treatment paradigm by starting ABC treatment 8 days after stereotaxic 6-OHDA lesion (Fig. 5a), when the akinetic behaviour was already manifested (Fig. 5b). We observed that ABC significantly improved the movement initiation time (Fig. 5c) as well as the number of adjusting steps (Fig. 5d) of chronically treated 6-OHDA-intoxicated rats. These results showed that ABC could also possess a possible symptomatic effect that improved the altered behaviour of rats already manifesting PD symptoms. To further strengthen these findings, we explored the early symptomatic effect of ABC after 2 days of treatment in the same intoxication paradigm (Fig. 5e). We observed that this short treatment with ABC was sufficient to improve the movement initiation time of 6-OHDA rats (Fig. 5f).

### ABC does not negatively interact with L-dopa

**Figure 6:**
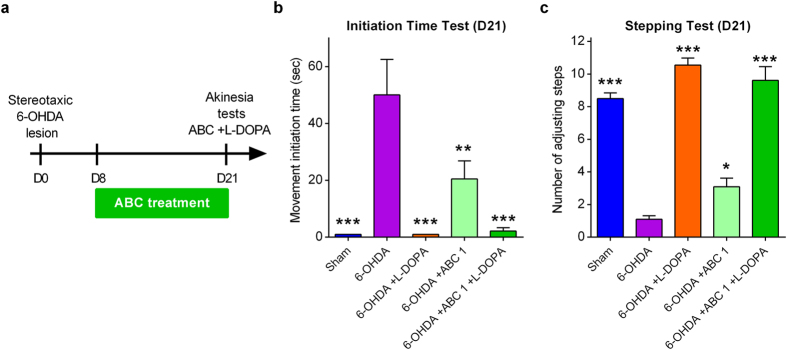
Absence of interaction between ABC and L-dopa on pharmacodynamics. Stereotaxically-lesioned 6-OHDA rats compared to Sham-injected animals. Animals were chronically treated p.o. twice a day with ABC from D8 after lesion until D20. On D21, animals were treated with ABC +L-dopa and then akinesia tests were performed (**a**). (**b,c**) Absence of interaction between L-dopa and ABC in the initiation time and stepping tests. L-dopa was used as reference drug. 6-OHDA or ABC did not have any effect in these behavioural tests when the control ipsilateral paw was assessed in the same rats in the initiation time and stepping tests. Values are mean ± s.e.m. *n* = 10 per condition except for L-dopa where n = 7. *P < 0.05, **P < 0.01, ***P < 0.001 versus 6-OHDA; ANOVA with Dunnett’s test. ABC 1: ACP 0.04 mg/kg + BCL 0.6 mg/kg. L-dopa: L-dopa 8 mg/kg + benserazide 12.5 mg/kg.

To assess whether there is any negative interaction between L-dopa and ABC on the akinetic behaviour, 6-OHDA-lesioned animals chronically treated with ABC from D8 to D21 were treated with ABC + L-dopa at the last day of treatment (Fig. 6a). We observed that in both the initiation time (Fig. 6b) and the stepping tests (Fig. 6c), animals treated with ABC + L-dopa were as responsive as animals treated with L-dopa only.

## Discussion

Despite all the therapeutic approaches developed after a half-century from discovery of L-dopa and its use to treat patients^33^, disease-modifying therapy for PD is at a standstill^34^. Several symptomatic options have been explored alone or in association with L-dopa. They demonstrated some clinical efficacy^35^, but their cost^36^ and marginal superiority compared to L-dopa limited the introduction of such approaches into clinical practice. In addition, in the quest for robust and effective treatments for replacement or combination to L-dopa, many compounds have proven their efficacy in animal models of PD^37^ but most of them failed in human clinical trials^38^. On the other hand, although L-dopa is the most cost-effective medication ever developed, it is a challenging drug to handle in PD patients and has significant limiting side effects^39^. Strikingly, it has been recently shown to induce dyskinesia in MPTP-intoxicated monkeys independently of a raise of striatal dopamine by an unravelled mechanism of action^40^, pointing out the complexity of the action of this molecule and the necessity for an alternative or supportive combination therapy to this drug.

By the time of diagnosis, the average loss of striatal dopamine is estimated to be superior to 60%^41^, and the degenerative process continues in the absence of an adequate intervention and despite L-dopa treatment. Therefore, there is a strong rationale and an urgent need for a disease-modifying neuroprotective therapy that should ideally also possess a symptomatic action to compensate the deficit already attained at the moment of diagnosis. We previously showed that a combination (ABC) of two already approved drugs, ACP and BCL, provides a neuroprotective activity in AD *in vitro* and *in vivo* models^18^ while restoring the levels of glutamate. Since AD and PD share several genetic, molecular and cellular features, we explored the possibility that ABC could be protective in *in vitro* and *in vivo* models of PD as well. As we have previously proved the synergistic activity of ABC in *in vitro* and *in vivo* models of AD^18^, we proceeded to test this combination as a new entity in animals. As preliminary step we have tested the individual drugs *in vitro* and demonstrated the advantage of combining them, providing the rational for testing ABC combination in animals.

We first demonstrated *in vitro* that ABC synergistically protected neurons from oxidative stress, a feature that is thought to be important in PD^21^. We then tested our drug combination in the 6-OHDA *in vitro* model of PD and observed that ACP and BCL positively interacted when combined, leading to an enhanced protective and synergistic response. Consequently, we used the 6-OHDA unilateral stereotaxic injection rat model to assess the efficacy of the combination *in vivo*. This model which is most commonly used for testing PD therapies, has the advantage of reproducibility and offers the benefit that each animal serves as its own control^29^,^38^. Furthermore, while the bilateral injection model could seem to mimic more obviously late stages in patients^42^, PD is characterized by unilateral to bilateral disease progression^43^ that is initially manifested by unilateral motor symptoms^44^. When tested in this model, ABC confirmed its potential to improve motor dysfunction. This improvement in function with ABC may be due to protection of dopaminergic neuronal cell bodies and their projections in 6-OHDA-treated rats. Consistent with this suggestion, we observed that ABC treatment, started before the induction of 6-OHDA lesion, attenuated loss of nigral dopaminergic neurons. Interestingly, we found that ABC treatment also increased tritiated mazindol binding in the striatum of lesioned rats, suggesting that our drug combination also protected dopaminergic nerve terminals. Another possible explanation of the anti-akinetic effect of ABC could include a symptomatic effect that improves the behaviour of rats with pre-existing lesions. In line with this hypothesis, we found that delayed ABC treatment, started when akinetic behaviour was already established, reversed the abnormal motor behaviour induced by 6-OHDA intoxication. Moreover, in the same paradigm, a treatment for 2 days with ABC was sufficient to partially restore normal motor behaviour. All these results taken together lead us to the conclusion that ABC treatment could exert both protective and symptomatic effects in the 6-OHDA-lesioned rat.

To date, the vast majority of PD patients are mostly treated by L-dopa. To further explore the feasibility of treating PD patients already medicated with L-dopa, with ABC, we sought to confirm that L-dopa and ABC did not negatively interact. We found that rats treated together with ABC and L-dopa were as responsive as L-dopa treated animals, excluding a potential negative pharmacodynamic interaction of such combination. This result could open a possibility for clinical testing of ABC by expanding the treatment to PD patients that are already treated with L-dopa. This observation does not exclude exploring further synergistic doses of ABC and L-dopa.

The exact molecular mechanism of action (MOA) of ABC is far from being elucidated, but ACP and BCL could synergistically act on relevant pathways increasing the efficacy of their combination, as observed in our *in vitro* experiments. On the one hand, ACP is widely used in relapse prevention of alcoholism by attenuating hyper-glutamatergic states observed in early abstinence. Although its precise MOA remains elusive, ACP is thought to influence glutamatergic transmission and to affect the excitatory/inhibitory balance distorted in ethanol addiction. Interestingly, ACP normalised GABA^45^ and glutamate^46^ levels in the ventral striatum of alcohol-dependent rats, both shown to be dramatically affected in the striatum of 6-OHDA intoxicated PD rats^47^. Moreover, ACP has also been shown to increase extracellular dopamine content in the nucleus accumbens^48^ and to positively modulate the mesolimbic dopaminergic system in the same cerebral structure^49^. BCL is a well characterised GABA_B_ receptor agonist clinically used for the symptomatic treatment of spastic movement disorders^50^. How BCL may interact with ACP in improving the akinetic behaviour in 6-OHDA intoxicated rats is still to be determined. It does not seem to affect dopamine release in the striatum^51^, but at the same time, it might modulate among others glutamate signalling by acting on presynaptic GABA_B_ receptors that can decrease the release of glutamate at glutamatergic synapses^52^. These previously described observations for ACP and BCL support the hypothesis that they might restore the glutamate/GABA signalling balance.

Dopamine deficiency triggers a cascade of functional changes in basal ganglia circuitry in PD^53^. Indeed, the essential pathophysiological state of PD is mediated through increased neuronal activity in the output nuclei of the basal ganglia (namely *globus pallidus* (GP) *pars interna* (GPi) and *substantia nigra pars reticulata* (SNr)) as a consequence of (i) a decreased GABAergic input from the putamen (direct striatal pathway) and (ii) an increased glutamatergic input from the subthalamic nuclei (indirect striatal pathway). These changes in activity are thought to lead to excessive inhibition of thalamocortical and brainstem motor systems^53^,^54^ (these pathophysiological changes in PD are reviewed in detail in^53^). Hence, a possible explanation for the observed symptomatic effect of ABC could be by its ability to reinstate GABAergic and glutamatergic inputs in the GPi/SNr, consequently normalising this activity and thus the functioning of thalamocortical and brainstem motor systems. Consistent with this view, Galvan *et al.* showed that microinjection of BCL in the GP of MPTP-treated monkeys decreased the firing rate of GPi neurons^55^. Future experiments are needed to shed light on the validity of such hypotheses and to understand the role and MOA of ABC in PD.

Collectively, our results demonstrate the potential value in the context of PD of (i) the combinational approach and (ii) the use for this purpose of previously existing drugs^56^. ACP and BCL have been used individually for a long time to treat other diseases and demonstrated only limited side effects despite the high dosages used. Moreover, the strong synergistic potency of the combination of drugs with well-known safety profiles will allow their use at lower doses, further decreasing the risk for potential undesirable events in a situation where a large number of new molecules failed in clinical trials due to safety concerns. Furthermore, several dozens of compounds have been shown to affect favourably PD-like pathology in experimental models, but very few reached even modest clinical efficacy. Thus, our goal at this step was essentially to provide through testing in reproducible preclinical models the rationale for further rapid translational testing of ABC in PD patients. At the same allometric low doses tested in animals in this study, we have already assessed this combination in AD patients in phase I/IIa (ClinicalTrials.gov Identifier: NCT02361424) clinical trial, in which no safety or tolerability issues were identified. Finally, we demonstrated that ABC possesses neuroprotective as well as symptomatic properties. Due to the complexity of PD and the use of several drugs in combination, the elucidation of its MOA will be of great importance, as soon as its efficacy is confirmed in patients.

In conclusion, combination of repurposed drugs may represent an efficient and valuable strategy to slow or stop the progression of PD, as we previously demonstrated for other neurological disorders such as AD^18^ or Charcot-Marie-Tooth disease 1A^57^,^58^.

## Methods

### Animals

Experiments in animals were performed according to the Council Directive of the European Union (2010/63/UE), minimising at best the number of animals used. In addition, French government approval of all protocols was granted, and ethics committees of Syncrosome, NeuroSys and Neuronexperts gave their permission to carry out all animal studies. Male Wistar rats (Janvier, Saint Berthevin, France), 5 weeks old at the beginning of the study, were used for 6-OHDA intoxications. Free access to food and water was allowed for animals except during behavioural experiments. Cages were maintained in a controlled room (50–60% humidity, 23 ± 1 °C) with a 12 h light/dark sequence. For *in vitro* studies, female Wistar rats (Janvier) were killed by cervical dislocation and E15 embryos were removed for neuron isolation for cell culture.

### Drugs

(RS)-baclofen (BCL), acamprosate calcium (ACP), riluzole (RIL), 6-hydroxydopamine (6-OHDA), L-3,4-dyhydroxyphenylalanine (L-dopa) and benserazide were provided by Sigma Aldrich. Brain-Derived Neurotrophic Factor (BDNF) was from PAN Biotech (Aidenbach, Germany). For *in vitro* studies, drugs were solubilized in distilled water and prepared before each administration. ACP, BCL, ABC, BDNF and RIL dissolved in 0.1% dimethylsulfoxide (DMSO) were added 1 h before oxidative stress induction or 6-OHDA intoxication. For *in vivo* experiments, ACP, BCL and ABC were dissolved in distilled water. 6-OHDA, L-dopa and benserazide were dissolved in 0.9% NaCl containing 0.1% ascorbic acid.

### Cell cultures

#### OGD model

Oxidative stress induction and treatments were validated and carried out at Neuronexperts laboratories (Marseille, France). Rat cortical neurons were cultured as described before^59^. Briefly, pregnant Wistar female rats were killed by cervical dislocation and the cortices of E15 embryos were dissected and mechanically dissociated. Cells were then trypsinised for 20 min at 37 °C and suspended after washing in a defined culture medium composed of Neurobasal medium supplemented with L-glutamine (0.2 mM), B27 (2%) (Invitrogen, France), 10 ng/mL of BDNF and 2% of penicillin/streptomycin (PS) solution. Cells were then seeded in poly-L-lysine pre-coated 96-well plates (Greiner, Courtaboeuf, France) and cultured at 37 °C in a humidified incubator (95% air/5% CO_2_). After 10 days of culture, cells were pre-treated for 1 h with ACP, BCL, ABC or RIL. One hour after drug incubation, the medium was removed and fresh medium without glucose and drugs was added. The cells were then transferred into an anaerobic incubator with 95% N_2_ and 5% CO_2_ at 37 °C. After 2 hours, 25 mM of D-glucose was added to culture medium and cells were transferred to a classic incubator. After 24 h of oxygen and glucose reperfusion, cells were fixed by a cold solution of alcohol/acetic acid during 5 minutes.

#### 6-OHDA model

Cultures, 6-OHDA intoxication and treatments were validated and performed at Neuronexperts and NeuroSys laboratories (Aix en Provence, France). Rat dopaminergic neurons were cultured as described by Visanji^60^. Briefly, the ventral midbrains obtained from 15-day-old rat embryos (Janvier, France) were dissected and trypsinised for 20 min at 37 °C. Cells were then washed and resuspended in a defined culture medium consisting of Neurobasal supplemented with B27 (2%), L-glutamine (2 mM) and 2% of PS solution and 10 ng/mL of BDNF and 1 ng/mL of Glial-Derived Neurotrophic Factor (GDNF). Cells were seeded in 96-well plates pre-coated with poly-L-lysine and maintained in a humidified incubator at 37 °C in 5% CO_2_ and 95% O_2_ atmosphere. After 6 days of culture, cells were pre-treated for 1 h with ACP, BCL, ABC or BDNF, then intoxicated with 6-OHDA (20 μM). After 48 h of intoxication with 6-OHDA, cells were fixed by a solution of 4% paraformaldehyde (Sigma). Drug treatment lasted until the end of the experiment.

#### Immuno-staining

After permeabilisation with saponin (Sigma), cells were blocked for 2 hours with PBS containing 10% goat serum, then incubated with mouse monoclonal primary antibody against Microtubule-Associated Protein 2 (MAP2, M4403, Sigma) for oxidative stress studies or against Tyrosine Hydroxylase (T1299, Sigma) for 6-OHDA cultures. These antibodies were revealed with Alexa Fluor 488 goat anti-mouse IgG (A-11001, Molecular probe). Hoechst (Invitrogen) counter-staining was used to visualise nuclei. For each condition, 10 pictures per well were taken using the InCell Analyzer^TM^ 1000 (GE Healthcare) with a 20× magnification.

### *In vivo* 6-OHDA lesion and treatment paradigms

Surgery was performed under ketamine (50 mg/kg) and xylazine (10 mg/kg). Animals received a unilateral injection of 12 μg of 6-OHDA dissolved in 6 μL of 0.9% sterile NaCl containing 0.1% ascorbic acid. The stereotaxic coordinates of the injection site in the left *substantia nigra pars compacta* (SNc) were: anteroposterior +2.2 mm, lateral +2.0 mm, dorsoventral +3 mm with the incisor bar at +5.0 mm above the interaural plane, according to the rat stereotaxic atlas by De Groot (1959). Animals were chronically treated *per os* (p.o.) by gastric gavage with ABC twice a day either for 16 days beginning one day before the 6-OHDA stereotaxic lesion (assessment of the neuroprotective effect of ABC) or for 2 or 14 days beginning 8 days after the 6-OHDA lesion (assessment of the symptomatic effect of ABC). For the interaction experiment, L-dopa treatment was associated with ABC on day 21. L-dopa and benserazide were injected i.p. 1 hour before functional tests.

### Behavioural analyses

Animals were assayed in a blind and random fashion. Treatment groups (*n* = 10 per group) were evenly represented in each cage. Acute 6-OHDA injection experiments and readouts were done and validated at Syncrosome facilities (Marseille, France). Animals that did not accomplish the minimal threshold requirements predefined in the protocol were excluded from analyses. The resulting number of animals used in each group is shown in the figure legends. Tests were performed on day 15 to assess the neuroprotective effect of ABC or on day 8 or 22 to assess the rapid symptomatic effect of ABC, 1 hour after L-dopa or 2 hours after vehicle or ABC treatment. Initiation time and stepping tests were also performed 7 days after 6-OHDA treatment to check that akinesia had already manifested when assessing the symptomatic effect of ABC.

#### Initiation time (IT) and stepping tests

Baseline values were obtained by performing the test three consecutive times for each limb and for each animal 2 days before the surgery. The animal was held by a trained technician in front of a plane surface. Only one of the two forelimbs was left free to move. For the IT test, the time necessary to initiate the movement toward the plane surface was recorded, using 180 s as break-off point^61^. For the stepping test, the animal was moved slowly forward during 5 s (0.9 m) and the number of adjusting steps was counted. Animals were tested for their affected contralateral paw as well as for their control ipsilateral one.

#### Cylinder test

After completion of the IT and stepping tests, each animal was scored for akinesia of the contralateral limb by using the cylinder test to assess forelimb asymmetry, in comparison to control rats. Animals were placed into a Plexiglas cylinder and videotaped for 15 min. The number of contacts made on the cylinder wall with the two paws at the same time (double contacts) was recorded and expressed as a percentage of the total number of contacts^62^. Only rats showing a minimum number of 7 wall contacts were included in the final analysis of the cylinder test.

### Histology

#### Tyrosine- hydroxylase (TH) neurons immuno-staining and quantification of TH cell loss

All animals were killed by lethal injection of pentobarbital and perfused with formaldehyde. For each animal, sections of the SNc were performed (from 4.8 to 5.8). Specific fluorescent staining of TH was obtained using a primary mouse anti-TH antibody (Merck Millipore, MAB318). The number of TH-positive neurons was counted in the ipsilateral and contralateral SNc on three sections per animal (anterior, medial and posterior) using an image analysis software.

#### *Evaluation of the loss of dopaminergic terminals in the striatum using*^*3*^ *H-mazindol staining*

All animals were killed by lethal injection of pentobarbital. Brains were quickly removed and then frozen in dry ice and stored at −80 °C. Coronal striatal sections were cut at −20 °C using a cryostat, rinsed and then incubated for 40 min with 15 nM [^3^H]-mazindol (DuPont, 17 Ci/mM). [^3^H]-sensitive photographic film (Kodak BioMax MS Film, Sigma) was apposed to the slides in X-ray cassettes and exposed at room temperature for 3 weeks. The level of [^3^H]-mazindol labelling was quantified by digitised image analysis from the film autoradiogram using a BIOCOM analysis system (Densirag, BIOCOM). Grey levels were converted to optical densities (ODs) using external standards. The mean OD value was determined from three sections for each animal after subtracting the background signal measured in corpus callosum on each section.

### Statistical analyses

Statistical tests were two-tailed and performed at 5% significance. In behavioural studies, sample sizes were defined on the basis of past experience on test variability. Distribution of data and group variations were assessed before analyses in order to guide our statistical choices. Statistical analyses were performed with Prism (http://www.graphpad.com/scientific-software/prism) and *R* (http://cran.r-project.org). We applied an Analysis of Variance (ANOVA) with Dunnett’s test for comparison of more than one experimental group against a reference.

Drug combination analysis was performed with *R* with calculation of a Combination Index (CI) recognized as the standard measure of combination effect that indicates a greater (CI < 1), lesser (CI > 1) or similar (CI = 1) effect than the expected additive effect^23^. CI was calculated according to two approaches based on different assumptions and adapted to different situations: (i) The Highest Single Agent approach^23^ is an effect based-approach reflecting the fact that the resulting effect of a drug combination is greater than the effects produced by its individual components. (ii) The Loewe additivity model^26^,^27^ is a dose-effect-based approach following the principle of dose equivalence and relies on the individual dose-effect curves.

## Additional Information

**How to cite this article**: Hajj, R. *et al.* Combination of acamprosate and baclofen as a promising therapeutic approach for Parkinson’s disease. *Sci. Rep.* **5**, 16084; doi: 10.1038/srep16084 (2015).
